# The American Medical Association

**Published:** 1850-07

**Authors:** 


### THE WESTERN JOURNAL.

THIRD SERIES.—VOL. VI.—NO. I.

LOUISVILLE, JULY 1, 1850.

### THE AMERICAN MEDICAL ASSOCIATION.

We had prepared, for this number, a copious report of the proceedings of the American Medical Association, and regret that want of space compels us to postpone its publication until August. This is a matter of some regret to us, for there are many points of interest in those proceedings, upon which we wish our readers to ponder well before the next meeting of the Association. Among these points of interest none are more important than the subject of medical education. While there was a great diversity of opinion among the members of the recent convocation, on the means of attaining an elevation of the standard of qualifications, there was no difference of sentiment as to the necessity.

There seems to be some plausibility in the idea that the means of medical education may be improved by prolonging the lecture terms, but we are disposed to doubt it. Students are not mere machines to have knowledge forced into them by the pressure of lectures alone. There must be time given for reading, reflection, writing on medical themes, and for as extended a field of observation as can be used in actual disease. We know of no other way to make practical physicians. Experience seems to have fixed upon four months, or their equivalent as the proper term of a course of lectures, and the whole of the remaining eight months of the year can be more profitably employed in other resources of medical improvement. Medical lectures are an excellent means of study, in their proper place, but there is such a thing as having too much even of something good. If medical lectures are up to the mark of true excellence, four months of devotion to them on the part of the teachers and pupils are sufficient, if they have not that quality of excellence, the lengthening of them cannot impart any additional goodness to them, but rather the reverse. Some medical teachers can impart more knowledge in four months than others can in twelve months, and we should prefer the short term to the long-winded professors.

Some one or two medical schools affirm that they have lengthened their lecture terms greatly to the advantage of the pupils, but we should rather see the signs of the advantages in the pupils, than hear of them from other parties. We thought we saw evident signs of distress in the very boasting of the lengthened terms, for the boasting was accompanied with an appeal to the Association to come to the help of the boasters against the mighty, and by way of remuneration to the Association for such help, the promise was tendered of a continuance in the paths marked out by that body. If the students find great advantages in the long term, they will not be slow to appreciate them, and they will be sure to avail themselves of those superior means. A great deal of this boasting, however, reminds us of country tavern signs, seen occasionally in the West, on which is announced in mishapen letters: "*Travellers taken in here.*" This might be slightly amended and read: *Students taken in here.*

The education of medical students is certainly an important subject for the consideration of the whole profession, and we shall rejoice in any feasible method for its improvement, but we are far from believing that all changes are necessarily improvements. B.

